# Direct economic costs related to antimicrobial resistance in bloodstream infections isolated from newborns in a perinatal hospital in Peru

**DOI:** 10.1093/inthealth/ihaf006

**Published:** 2025-01-30

**Authors:** Maria J Pons, Antonio M Quispe, Miguel Tirado, Gabriela Soza, Joaquim Ruiz

**Affiliations:** Universidad Cientifica del Sur, Villa el Salvador, Lima 15067, Perú; Universidad Señor de Sipán, Chiclayo 1502, Perú; Instituto Nacional Materno Perinatal de Lima, Cercado de Lima, Lima 15001, Perú; Instituto Nacional Materno Perinatal de Lima, Cercado de Lima, Lima 15001, Perú; Universidad Cientifica del Sur, Villa el Salvador, Lima 15067, Perú

**Keywords:** antibiotic resistance, bacteremia, costs, neonates, Peru

## Abstract

**Background:**

Antimicrobial resistance (AMR) has emerged as a priority for both public health and the global economy. Moreover, information on AMR is scarce, particularly in low/middle-income countries. We evaluated the direct economic cost of microorganisms and AMR.

**Methods:**

We performed a cross-sectional study to assess the economic costs of neonatal cases diagnosed with bacteremia at the Instituto Nacional Materno Perinatal in Lima, Peru, from January 2017 to June 2018. We used cost invoices calculated by the micro-costing bottom-up approach, as well as the strain identification and antimicrobial susceptibility data, to estimate the direct costs.

**Results:**

The average costs of bacteremia were US$349 (SD 403) for multidrug-resistant (MDR) strains and US$276 (SD 349) for non-MDR strains. Costs were higher for microorganisms associated with late-onset sepsis (LOS). We found that LOS, multidrug resistance and age were significantly associated with bloodstream infection (BSI) costs. Also, all microorganism groups were associated with increased costs, with the highest average costs for *Acinetobacter*, followed by *Pseudomonas*.

**Conclusions:**

In Peru, BSI costs are strongly associated with AMR. Furthermore, costs increase significantly with LOS, multidrug resistance and the patient's age. We urge health authorities to strengthen measures and strategies against the pressing threat of AMR.

## Introduction

In recent decades, antimicrobial resistance (AMR) has emerged as one of the most pressing threats to public health and the global economy. This problem is so severe that the WHO has identified it as a worldwide priority and has issued a list of antibiotic-resistant bacteria that guide research and development of new antimicrobials.^1,2^

In 2019, predictive statistical models estimated 4.95 (3.62–6.57) million deaths associated with bacterial AMR, including 1.27 (95% UI 0.911–1.71) million deaths attributable to bacterial AMR in 204 countries.^3^ In addition, one report estimated that 10 million deaths will be attributed to AMR by 2050.^4^ It is highlighted that the burden of AMR falls particularly heavily on low/middle-income countries (LMIC), where AMR causes a higher number of deaths, with a higher percentage of young people.^5^ Specifically, direct deaths from AMR among children were 40.8 per 100 000 children aged <5 y in LMIC.^5^

Regarding the economic costs related to AMR, a prediction of US$100 trillion of the world's economic output will be lost.^4^ These economic costs are mainly associated with increased treatment costs and more extended hospital stays, which would translate into increased morbidity and mortality, with a particular impact on the fragile healthcare systems of LMIC.^4,6^

Several resistant bacteria have been increasingly involved in infectious diseases in humans, specifically, *Enterococcus* spp., *Staphylococcus aureus, Klebsiella pneumoniae, Acinetobacter baumannii, Pseudomonas aeruginosa* and *Enterobacter* spp. (ESKAPE).^1,7^ In addition to increased morbidity and mortality, antimicrobial-resistant ESKAPE bacteria are associated with significantly increasing healthcare costs.^7^

More evidence on AMR’s economic outcomes and health impact is needed, which is a major obstacle to understanding its actual effects.^8^ Regarding the economic impact, studies at the local level are recommended, as it is necessary to have a view of AMR at the regional level to provide a more realistic and contextualized picture of the costs of AMR, as they can consider localized epidemiological priorities and health service standards.^6,9^ In Peru, no information on economic costs and AMR related to infections has been established. Thus, this study aimed to evaluate the economic cost associated with microorganisms and antibiotic resistance patterns in bacteremia among newborns with bloodstream infection (BSI) from a maternal perinatal hospital in Lima, Peru.

## Material and methods

### Study design and population

We performed a cross-sectional study to assess the direct economic costs of neonates with positive bacteremia diagnosed at the National Maternal Perinatal Institute (INMP) of Lima from January 2017 to June 2018.^10^ When microorganisms causing bacteremia were isolated within the first 72 h of life, the condition was classified as early-onset sepsis (EOS), whereas cases diagnosed after the first 72 h were classified as late-onset sepsis (LOS).

The inclusion criteria for the study were newborns (<28 d of life) with a diagnosis of neonatal sepsis confirmed with positive bacteremia analyzed by blood culture. In addition, to avoid reporting bias, exclusion criteria included contaminated blood cultures and samples collected from patients receiving antibiotic treatment during sample collection.^10^

The INMP is the largest maternity reference hospital in Peru (>20 000 births annually) with full insurance coverage. Peru's healthcare system is mixed, comprising public, social security and private sectors. The Ministry of Health provides services to the majority of the population through a network of hospitals and health centers, focusing on primary care and maternal-child health, including the INMP. The social security system (EsSalud) covers formally employed workers and their dependents, while the armed forces and police have their own parallel health systems. Private clinics and hospitals mainly serve individuals with private insurance or those who can pay out of pocket. This fragmented structure often challenges coordinated efforts to tackle public health issues such as AMR, highlighting the need for strong policies and integrated strategies.

### Strain identification and antimicrobial susceptibility

Blood samples were incubated in an automated blood culture system (BD BACTEC) for 7 d before reporting no growth. A Gram stain and subcultures in selective media were performed to identify causative agents, both according to conventional microbiology protocols.^10^ The antimicrobial susceptibility was evaluated by the disk-diffusion method and the results were interpreted according to the Clinical and Laboratory Standards Institute guidelines.^11^ Bacteria resistant to at least one antibiotic in three or more drug classes were multidrug-resistant (MDR) according to criteria reported by Magiorakos et al.^12^

### Economic costs

During the study period, we assessed the invoices associated with hospital stays of neonates with positive bacteremia. These costs did not include the cost of procedures but included the direct cost of materials and treatments received during the stay. We did so because procedure costs often fall under broader, fixed overhead expenditure or bundled service charges that are not easily disaggregated for micro-costing analyses. By contrast, consumables (e.g. medications, materials, supplies) directly linked to each patient can be itemized and attributed with greater precision. The costs were converted to US$ at the average exchange rate during the study period (3.30 Peruvian sol (PEN) per 1 US$).

## Results

### Characteristics of population

A total of 288 samples were included: 225 (71.1%) among MDR isolates and 63 (21.9%) non-MDR. Among these, 197 (68.4%) were isolated among LOS, and 91 (31.6%) were EOS. Also, 56.9% were female; most samples came from inpatients, with only 5.2% from outpatients (Table 1).

**Table 1.** General characteristics of the study subjects

	EOS		LOS		Total	
Characteristic	Non-MDR N= 34	MDR N= 57	Non-MDR N= 29	MDR N= 168	Non-MDR N= 63	MDR N= 225
Age, d (mean±SD)	1.6±1.0	1.7±1.0	11.9±6.5	12.5±7.3	6.3±6.8	9.7±7.9
Male gender	16 (47.1%)	25 (43.9%)	15 (51.7%)	68 (40.5%)	31 (49.2%)	93 (41.3%)
Outpatient	2 (5.9%)	0 (0.0%)	3 (10.3%)	10 (6.0%)	5 (7.9%)	10 (4.4%)
Bacteria group						
*Staphylococcus*	26 (76.5%)	45 (79.0%)	16 (55.2%)	112 (66.7%)	42 (66.7%)	157 (69.8%)
*Enterobacterias*	0 (0.0%)	8 (14.0%)	5 (17.2%)	32 (19.1%)	5 (7.9%)	40 (17.8%)
*Acinetobacter*	0 (0.0%)	1 (1.8%)	0 (0.0%)	16 (9.5%)	0 (0.0%)	17 (7.6%)
*Streptoccocus*	2 (5.9%)	2 (3.5%)	2 (6.9%)	5 (3.0%)	4 (6.4%)	7 (3.1%)
*Enteroccocus*	5 (14.7%)	1 (1.8%)	3 (10.3%)	2 (1.2%)	8 (12.7%)	3 (1.2%)
*Stenotrophomonas*	1 (2.9%)	0 (0.0%)	2 (6.9%)	0 (0.0%)	3 (4.8%)	0 (0.0%)
*Pseudomonas*	0 (0.0%)	0 (0.0%)	1 (3.4%)	1 (0.6%)	1 (1.6%)	1 (0.4%)
Bacteria species						
*CoNS*	23 (67.7%)	40 (70.2%)	13 (44.8%)	96 (57.1%)	36 (57.1%)	136 (60.4%)
Other *Staphylococcus* spp.	3 (8.8%)	5 (8.8%)	3 (10.3%)	16 (9.5%)	6 (9.5%)	21 (9.3%)
*E. coli*	0 (0.0%)	5 (8.7%)	2 (6.9%)	12 (7.1%)	2 (3.2%)	17 (7.6%)
*Acinetobacter* spp.	0 (0.0%)	1 (1.8%)	0 (0%)	16 (9.5%)	0 (0.0%)	17 (7.6%)
*Klebsiella* spp.	0 (0.0%)	2 (3.5%)	3 (10.3%)	9 (5.4%)	3 (4.8%)	11 (4.9%)
*Serratia* spp.	0 (0.0%)	1 (1.8%)	0 (0%)	11 (6.6%)	0 (0%)	12 (4.9%)
*Enterococcus* spp.	5 (14.7%)	1 (1.8%)	3 (10.3%)	2 (1.2%)	8 (12.7%)	3 (1.3%)
*Streptococcus* spp.	2 (5.9%)	2 (3.5%)	2 (6.9%)	5 (3.0%)	4 (6.4%)	7 (3.1%)
*S. maltophilia*	1 (2.9%)	0 (0%)	2 (6.9%)	0 (0.0%)	3 (4.8%)	0 (0.0%)
*P. aeruginosa*	0 (0.0%)	0 (0%)	1 (3.4%)	1 (0.6%)	1 (1.6%)	1 (0.4%)
Total costs, US$ (mean±SD)	130±140	152±204	447±436	416±431	276±349	349±403

*CoNS*: coagulase-negative *Staphylococci*; EOS: early-onset sepsis; LOS: late-onset sepsis; MDR: multidrug-resistant.

The most frequently isolated microorganisms belonged to the genus *Staphylococcus* (199; 69.1%), followed by 45 (15.6%) of the Enterobacteria group (*Escherichia coli, Klebsiella* spp. and *Serratia marcescens*). The coagulase-negative staphylococci (*CoNS*) group represented 59.7% of isolates (Table 1).

The average economic costs for 288 bacteremia cases were US$349 (SD 403) for MDR and US$276 (SD 349) for non-MDR. Costs were higher in treating strains related to LOS, both in MDR and non-MDR isolates (US$416 [SD 431] and US$447 [SD 436], respectively) (Table 1).

### Economic costs associated with bacteria genus

**Figure 1.**
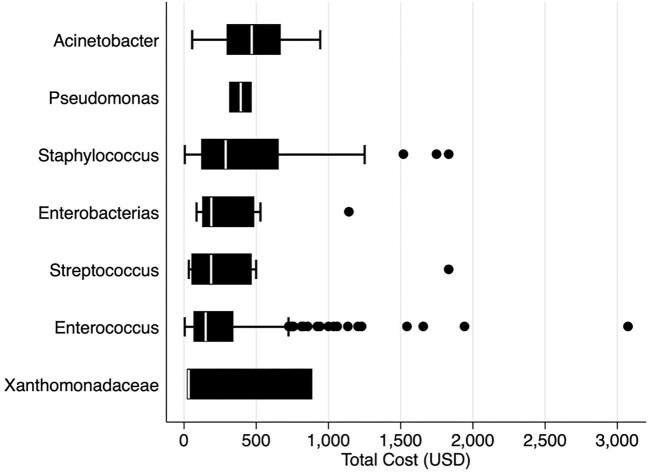
Total costs (US$) according to groups of microorganisms isolated.

The average costs per infection, according to microorganisms, were variable. The *Acinetobacter* genus has the highest cost (US$ 462.81 [SD 247.71]), followed by *Pseudomonas* (US$392.38 [SD 100.46]) (Figure 1).

### Economic costs associated with LOS vs EOS infection

**Figure 2.**
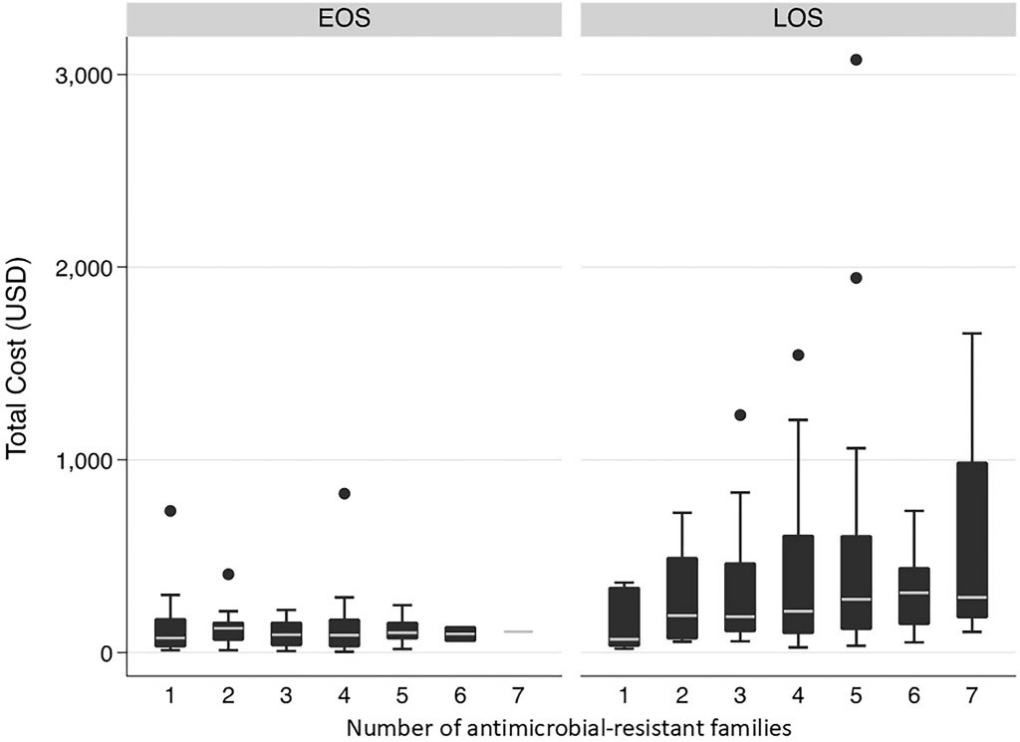
Total costs of microorganisms resistant to different antibiotics families (one to seven), in LOS and EOS. EOS: early-onset sepsis; LOS: late-onset sepsis.

Except for LOS-associated microorganisms with resistance to one family of antimicrobials, LOS-associated microorganisms had higher costs compared with EOS-associated microorganisms, even those resistant to several families of antimicrobials (Figure 2). In addition, the average economic cost increased as the number of antibiotic-resistant families increased.

### Cost-associated factors

In the regression analysis, we identified that LOS, multidrug resistance and age were significantly associated with economic costs related to infection. In our multivariable regression analysis, we observed that the costs were mainly higher among cases with LOS compared with those with EOS (incidence rate ratio [IRR] 2.92, CI 2.87 to 2.98) among MDR microorganisms (IRR 1.26 [1.24 to 1.29]) and age (IRR 1.04 [1.03 to 1.04]), with p<0.001, respectively. Also, all the microorganism groups were associated with economically increased costs compared with the *Staphylococcus* group (p<0.0001), except for *Stenotrophomonas*. The multivariate model was performed, adjusting for age and gender, and the association between LOS (adjusted IRR 2.94, CI 2.88 to 3.00) and the different microorganisms’ groups was maintained, except for *Stenotrophomonas* and *Pseudomonas* (Table 2).

**Table 2.** Regression analysis for the total cost among newborns with BSI

Associated factor	IRR (95% CI)	p	aIRR (95% CI)	p
LOS	2.92 (2.87 to 2.98)	<0.001	2.94 (2.88 to 3.00)	<0.001
MDR	1.26 (1.24 to 1.29)	<0.001	0.95 (0.94 to 0.97)	<0.001
Outpatient	0.37 (0.35 to 0.38)	<0.001	0.33 (0.31 to 0.34)	<0.001
Bacteria group				
*Staphylococcus*	Ref		Ref	
*Enterobacteria*	1.61 (1.59 to 1.64)	<0.001	1.38 (1.36 to 1.40)	<0.001
*Acinetobacter*	1.60 (1.56 to 1.64)	<0.001	1.22 (1.19 to 1.25)	<0.001
*Streptoccocus*	1.21 (1.17 to 1.25)	<0.001	1.34 (1.30 to 1.39)	<0.001
*Enteroccocus*	1.17 (1.13 to 1.21)	<0.001	1.14 (1.10 to 1.18)	<0.001
*Stenotrophomonas*	1.08 (1.01 to 1.15)	0.016	0.97 (0.91 to 1.03)	0.327
*Pseudomonas*	1.35 (1.26 to 1.45)	<0.001	0.97 (0.90 to 1.04)	0.350
Male gender	0.97 (0.96 to 1.29)	<0.001		
Age (d)	1.04 (1.03 to 1.04)	<0.001		

aIRR: adjusted incidence rate ratio; BSI: bloodstream infection; IRR: incidence rate ratio; LOS: late-onset sepsis; MDR: multidrug-resistant.

## Discussion

The present study provides the direct economic costs associated with BSI in neonates at a maternal and perinatal institution in Lima, Peru. It highlights that these costs were associated with microorganism groups, with the average cost higher for non-fermenting bacilli such as *Acinetobacter* spp. and *Pseudomonas* spp. In addition, costs also increased for microorganisms associated with LOS in general and for the increment of antibiotic-resistance families.

The previous study conducted in the same institution related to MDR microorganisms associated with BSI infections reported that the multidrug resistance rate among newborns with BSI was high, especially in the LOS group.^10^ Thus, it can be reported that multidrug resistance impacts the direct economic costs of treatment of sepsis in neonates at the INMP. In our study, we have reported the association of multidrug resistance with an increase in economic costs (IRR 1.26. [1.24 to 1.29]). These results are consistent with previous studies that reported a more significant economic burden in the MDR compared with the non-MDR group in microorganisms associated with urinary tract infections.^13^ Thus, these costs were significantly associated with more extended hospital stays, antibiotic use and catheter use.^13,14^ Furthermore, AMR is associated with more significant early clinical failure,^15^ often related to incorrect empirical treatment; for example, in a study in Thailand, a country with a gross domestic product per capita similar to Peru, 29.9% of BSI received inappropriate empiric treatment.^16^ Moreover, in BSI, in particular, resistance to three generations of cephalosporins resulted in a mean excess length of 1.78 d in hospital stay, and the probability of a positive excess length of stay was 95%.^17^

The results showed that, for both EOS and LOS, microorganisms from the *CoNS* group were the most frequently isolated. Traditionally, this microorganism was considered a contaminant and non-pathogenic, but in recent decades, it has been recognized as an important human pathogen, being the most isolated in NICU.^18,19^ Although the most common bacterial was *CoNS*, this study shows that, on average, the genus *Acinetobacter* spp. offered the highest direct costs. Furthermore, all *Acinetobacter* were MDR. Previous research in this institution placed this bacterium in the top two causative agents of BSI related to LOS in newborns.^10^ The Carbapenem-resistant *A. baumannii* has recently been included in the critical group of bacterial pathogens of public health importance that pose a significant threat to human health, prioritizing research and development efforts for new antimicrobial treatments, according to the WHO.^20^ Thus, its presence in the neonatal group is of great concern, as is Enterobacterales, which is resistant to third-generation cephalosporins and carbapenems.^20^

Although our study has not analyzed specific resistance to a family of antibiotics such as extended-spectrum beta-lactamases (ESBLs) or carbapenemases, some studies relate the presence of resistance to increased costs.^15,21^ Thus, carbapenemase-producing Enterobacteriaceae (CPE) presented double hospitalization costs compared with non-CPE and required more long-term care facilities and outpatient parenteral antibiotic therapy regarding resource consumption.^15^ Also, for patients with BSI, those with ESBL-producing *E. coli* and *Klebsiella* spp. incur higher costs than those with non-ESBL *E. coli* and *Klebsiella* spp., mainly due to different rates of effective empirical antimicrobial treatment and differences in the length of hospital stay.^21^

It is important to note that multidrug resistance entails a significant economic cost to families. Our findings were that the average cost of MDR strains (direct medical cost) is US$349; this value is high considering that the minimum salary in Peru in 2019 was 930 soles, which is equivalent to approximately US$281.82. AMR generates an actual cost that affects the economic circumstances of each patient.^22^ Data on economic costs in AMR are scarce, particularly in LMIC. An example is the additional cost of €100 (95% CI 78 to 125) related to direct medical costs (hospital stay and antibiotic drugs) attributable to ESBL production in Senegal.^23^ Large differences are observed between the economic amounts in the different countries and studies. In Spain, the mean cost associated with bacteremia for admissions was €25 891,^24^ while in India, patients with hospital-acquired bacteremia experienced costs that were significantly higher (mean: US$4818) than those for controls.^25^ These differences were mainly associated with the different methodologies for estimating the additional costs of hospital-acquired infections.^26^

MDR organisms are on the rise worldwide as an increasing threat to public health, and they are also reducing the treatment effectiveness of available antimicrobials.^27^ Unfortunately, the weakness of the health management systems of LMIC hinders the population's access to state-of-the-art antibiotics and new treatments.^28^

As a limitation, it is important to note that this study has only evaluated the direct costs, and other confounding factors that could have influenced the results cannot be excluded. In addition, apart from the direct costs, it would be very interesting to evaluate the indirect costs associated with productivity loss to health systems and the general population, as well as affecting the morbidity and mortality of infected patients by calculating wages lost due to death or sick leave.^29^ Additionally, we evaluated only a single hospital institution, which does not necessarily represent the national reality.

The current study, as well as others investigating the economic impact of AMR on different health systems and, moreover, studies on the cost-effectiveness of applying vaccines to control AMR, are important for making political decisions on allocating resources for this priority problem.^30^

## Conclusions

This study shows the direct economic costs associated with BSI in neonates, according to association with EOS and LOS, AMR, and the isolated microorganism's group. These data can be used to begin to estimate the number of AMR cases in Lima regarding BSI in neonates, to help define strategies for its prevention and control.

## Data Availability

Data supporting the findings of this study are available upon reasonable request from the corresponding author. Restrictions apply to the availability of these data due to ethical considerations.
